# Association of Neighborhood-Level Socioeconomic Measures With Cognition and Dementia Risk in Australian Adults

**DOI:** 10.1001/jamanetworkopen.2022.4071

**Published:** 2022-03-25

**Authors:** Matthew P. Pase, Ella Rowsthorn, Marina G. Cavuoto, Alexandra Lavale, Nawaf Yassi, Paul Maruff, Rachel F. Buckley, Yen Ying Lim

**Affiliations:** 1 Turner Institute for Brain and Mental Health, Monash University, Clayton, Victoria, Australia; 2 Harvard T.H. Chan School of Public Health, Harvard University, Boston, Massachusetts; 3 Department of Medicine and Neurology, Melbourne Brain Centre at The Royal Melbourne Hospital, University of Melbourne, Parkville, Victoria, Australia; 4 Population Health and Immunity Division, The Walter and Eliza Hall Institute of Medical Research, Parkville, Victoria, Australia; 5 Cogstate Ltd, Melbourne, Victoria, Australia; 6 Melbourne School of Psychological Sciences, University of Melbourne, Melbourne, Victoria, Australia; 7 Department of Neurology, Massachusetts General Hospital and Harvard Medical School, Boston, Massachusetts; 8 Center for Alzheimer Research and Treatment, Department of Neurology, Brigham and Women’s Hospital, Boston, Massachusetts

## Abstract

**Importance:**

Up to 40% of dementia cases are potentially preventable; therefore, it is important to identify high-risk groups to whom resources could be targeted for maximal impact in preventing late-life dementia. The association of neighborhood-level socioeconomic status (SES) with cognition and dementia risk is not well known, particularly in midlife when late-life dementia may still be preventable through established interventions, such as blood pressure management.

**Objective:**

To examine whether neighborhood-level SES is associated with differences in cognitive performance and dementia risk scores.

**Design, Setting, and Participants:**

This cross-sectional study analyzed data collected between November 17, 2016, and April 14, 2020, from 4656 participants in the longitudinal population-based Healthy Brain Project cohort. This large online cohort comprised community-dwelling individuals geographically dispersed across Australia. Participants were aged 40 to 70 years without dementia or other major neurological conditions.

**Exposures:**

Neighborhood-level SES was computed by matching participants’ residential addresses to the Australian Bureau of Statistics Index of Relative Socio-economic Advantage and Disadvantage (IRSAD). Postcodes provided by each participant were used to derive an IRSAD score that ranked participants according to deciles of neighborhood-level SES (range, 1-10, with higher deciles indicating greater socioeconomic advantage); neighborhoods in deciles 1 to 7 were considered to have low or intermediate SES, and neighborhoods in deciles 8 to 10 were considered to have high SES.

**Main Outcomes and Measures:**

Dementia risk estimated using the dementia risk score from the Cardiovascular Risk Factors, Aging, and Incidence of Dementia (CAIDE) tool (n = 4656) and cognitive composite scores for memory and attention measured by the Cogstate Brief Battery (n = 2181).

**Results:**

Of 4656 participants (mean [SD] age, 56.1 [7.2] years; 3445 women [74.0%]), 2688 individuals (57.7%) lived in areas with high neighborhood-level SES (IRSAD decile ≥8), and 1968 (42.3%) lived in areas with low or intermediate neighborhood-level SES (IRSAD decile <8), with 1263 individuals (27.1%) residing in rural or regional areas. A total of 6 participants (0.1%) identified as African, 121 (2.6%) as Asian, 57 (1.2%) as Indigenous Australian, 24 (0.5%) as Latin American, 9 (0.2%) as Pacific Islander, 3671 (78.8%) as White or European, and 768 (16.5%) indicated other race (not specified). Each decile unit increase in neighborhood-level SES was associated with a lower CAIDE dementia risk score after adjustment for race and rurality (β [SE] = −0.070 [0.019]; *P* = .004). Each decile unit increase was also associated with better memory (β [SE] = 0.022 [0.006]; *P* = .006) but not with better attention (β [SE] = 0.009 [0.007]; *P* = .34), as measured by Cogstate Brief Battery composite *z* scores after adjustment for age, sex, race, years of education, and rurality. When comparing memory performance between individuals with IRSAD scores higher and lower than decile 8, neighborhood-level SES interacted with age (*F*_1-2171_ = 6.33; *P* = .02) and CAIDE dementia risk scores (*F*_1-2173_ = 4.02; *P* = .08). Differences in memory between neighborhood-level SES categories were larger among participants who were older and had a higher risk of dementia.

**Conclusions and Relevance:**

In this study, higher neighborhood-level SES was associated with better memory and lower dementia risk scores. These results suggest that efforts to lower dementia risk factors in disadvantaged areas are needed to curtail the increasing burden of dementia and that inclusion of individuals living in areas with lower SES in research on dementia is warranted to improve understanding and potential interventions.

## Key Points

**Question:**

Is neighborhood-level socioeconomic status (SES) associated with differences in cognition and dementia risk scores?

**Findings:**

In this cross-sectional study of 4656 Australian adults aged 40 to 70 years enrolled in the population-based Healthy Brain Project cohort, higher neighborhood-level SES was associated with better memory and lower dementia risk scores.

**Meaning:**

This study’s findings suggest that dementia research would benefit from including participants living in areas with lower SES to better understand relevant factors and potential interventions.

## Teaser

This cross-sectional study examines whether neighborhood-level socioeconomic status is associated with differences in cognitive performance and dementia risk scores among Australian adults aged 40 to 70 years enrolled in the Healthy Brain Project.

## Introduction

Increasing life spans have led to a higher prevalence of dementia. However, cognitive impairment does not appear to affect all communities equally. Dementia risk and prevalence have been found to vary by geographic location and neighborhood characteristics,^1,2^ even within developed nations.

As reported previously,^3^ living in areas with lower neighborhood-level socioeconomic status (SES) is associated with worse cognition among older adults.^4,5,6,7,8,9,10,11^ Similar to other health outcomes that differ by neighborhood-level SES, the mechanisms underlying these findings are multifactorial. They are likely to reflect interactions between various psychological, social, and environmental factors, including crime, safety,^12^ social disorder,^13^ local resources^14^ (including places for health care, education, work, leisure, and social engagement), air and noise pollution,^15^ access to park land, neighborhood walkability,^16,17^ and psychosocial resources.^18^ These factors, in turn, can impact long-term health outcomes and health-promoting behaviors (such as outdoor activity and exercise^19^ as well as social^14^ and economic engagement), social cohesion, and stress.^13^

Given that up to 40% of dementia cases are potentially preventable through risk factor modification,^20^ it is important to identify high-risk groups for whom targeted interventions addressing dementia risk factors and the associated challenges in addressing those risk factors could have maximal impact for mitigating late-life dementia risk. Therefore, further research is needed to ascertain whether dementia risk factors and cognition differ by neighborhood-level SES.^3^ It is particularly important to establish associations between neighborhood-level SES, cognition, and dementia risk in midlife because (1) the associations between several modifiable dementia risk factors and brain health are greatest when risk factors are measured in midlife^21^ and (2) persons in midlife are a good target for dementia prevention initiatives because late-life dementia, which has a long preclinical phase lasting several decades, may still be preventable. However, most relevant studies have examined the associations between neighborhood-level SES and cognition in older cohorts.^3^

The Healthy Brain Project (HBP)^22^ is an ongoing prospective cohort study of dementia-free adults aged 40 to 70 years at enrollment, enriched for family history of dementia (via advertisement aimed at recruiting those with family history or suspicion of family history), who may be at increased risk of cognitive decline but are unlikely to present with cognitive impairment or dementia.^23^ Given the online nature of the study, a large number of geographically diverse participants across Australia were recruited; 27% of participants reported a residential address in rural or regional Australia. This cohort provided an opportunity to examine associations between neighborhood-level SES, cognition, and dementia risk, measured using the well-validated Cardiovascular Risk Factors, Aging, and Incidence of Dementia (CAIDE) dementia risk score.^24^ We hypothesized that higher neighborhood-level SES would be associated with better cognition and lower dementia risk scores. We also explored whether age, dementia risk, or years of education moderated any differences in cognition between groups living in areas with higher and lower neighborhood-level SES.

## Methods

### Participants

A total of 4656 participants enrolled in the HBP were included in this cross-sectional study, with data collected between November 17, 2016, and April 14, 2020. The Monash University Human Research Ethics Committee approved the HBP; this approval pertained to all analyses of HBP data, including the current cross-sectional analysis. All participants provided written informed consent. This study followed the Strengthening the Reporting of Observational Studies in Epidemiology (STROBE) reporting guideline for cross-sectional studies.

The process of recruitment and enrolment has been described previously.^23^ In brief, participants were eligible for enrollment if they were aged 40 to 70 years, currently resided in Australia, and were fluent in English. Participants were excluded if they self-reported having a major neurological disease (including dementia) or insult, having a major psychiatric condition, or receiving any Therapeutic Goods Administration–approved medication for the treatment of Alzheimer disease. Participants were self-referred and recruited through various sources, such as social media posts, word of mouth, and advertisements. The present analyses were cross-sectional and included participants who completed a baseline assessment between 2016 and 2020.

### Measures

#### Neighborhood-Level SES

The Australian Bureau of Statistics developed the Index of Relative Socio-economic Advantage and Disadvantage (IRSAD) using data from the 2016 census. The IRSAD ranks neighborhoods throughout Australia from most disadvantaged to most advantaged based on the SES characteristics of the area.^25^ The IRSAD is derived from a combination of 11 socioeconomic variables, such as income, years of education, unemployment rates, occupational skills, disability, vehicle ownership, internet connection, family structure (eg, 1 parent with a dependent), and housing arrangements (more details are available in eMethods and eTable 1 in the Supplement). We used the postcode provided by each participant to derive an IRSAD score that ranked participants according to deciles of neighborhood-level SES (range, 1-10, with higher deciles indicating greater socioeconomic advantage); neighborhoods in deciles 1 to 7 were considered to have low to intermediate SES, and neighborhoods in deciles 8 to 10 were considered to have high SES.

#### Urban vs Rural Location and Personal SES

We also matched postcodes to data from the Australian Bureau of Statistics to categorize each participant’s location as urban (ie, metropolitan) vs rural or regional according to Accessibility/Remoteness Index of Australia categories; this index categorizes locations using measures of road distance from a given location to the nearest service centers based on population.^26^ We also categorized personal SES according to the Australian Socioeconomic Index 2006^27^ (eMethods in the Supplement). Each occupational group has a corresponding value on the Australian Socioeconomic Index 2006, which was used as the measure of personal SES; higher occupational scores reflect higher personal SES (eg, the occupational score for cleaners and laundry workers is 20.4, and the occupational score for accountants is 83.7).

#### Cognition

Participants completed the Cogstate Brief Battery using an unsupervised online platform. The acceptability, usability, and validity of the Cogstate Brief Battery in unsupervised settings has been reported previously.^28,29,30^ The battery consists of 4 tasks: detection, identification, one card learning, and one-back. These tests have been described in detail previously.^31,32^ In brief, the detection task is a simple reaction time exercise measuring psychomotor function, and the identification task is a choice reaction time exercise measuring visual attention. The primary outcome for the detection and identification tasks was reaction time in milliseconds, with lower scores indicating faster task completion. The one card learning task is a continuous visual recognition task set within a pattern separation model, and the one-back task is a working memory exercise. The primary outcome for the one card learning and one-back tasks was the proportion of correct responses, normalized using an arcsine square root transformation, with higher scores indicating better performance.

 The 4 tasks were combined into 2 composite measures assessing memory and attention. The attention composite was computed by standardizing and calculating the mean of scores from the detection and identification tasks. Because the detection and identification tasks are speeded measures, they were reverse scored so that negative values reflected worse performance. The memory composite was computed by standardizing and calculating the mean of scores from the one card learning and one-back tasks. All task scores were standardized using the baseline mean (SD) score of the entire sample. Higher scores on both the memory and attention composites indicated better performance.

#### General Health and Overall Dementia Risk

Participants self-reported their date of birth, sex, race, years of education, residential address, height and weight (used to compute body mass index, which was calculated as weight in kilograms divided by height in meters squared), blood pressure level, current cigarette smoking, and history of hypercholesterolemia and diabetes. To characterize the sample, we asked participants to complete the Hospital Anxiety and Depression Scale (score range, 0-21 points for each subscale [anxiety or depression], with subscale scores >8 points indicating anxiety or depression),^33^ the RAND 36-Item Short Form Survey (score range, 0-100 points, with higher scores indicating more favorable state of health),^34^ and the Perceived Stress Scale (score range, 0-40 points, with higher scores indicating higher perceived stress levels).^35^ Dementia risk was estimated using the CAIDE dementia risk score (see eTable 2 in the Supplement). The original CAIDE dementia risk score combines age, years of education, sex, history of hypercholesterolemia, history of hypertension, physical activity, and body mass index. Possible scores ranged from 0 to 15 points, with higher scores indicating a higher risk of dementia.^24^ To estimate dementia risk associated with modifiable dementia risk factors only, we also created a modified CAIDE dementia risk score that included only those risk factors that were readily modifiable, including physical activity, history of hypercholesterolemia, history of hypertension, and body mass index. Modified CAIDE scores ranged from 0 to 7 points, with higher scores indicating higher dementia risk.

### Statistical Analysis

**Figure 1.**
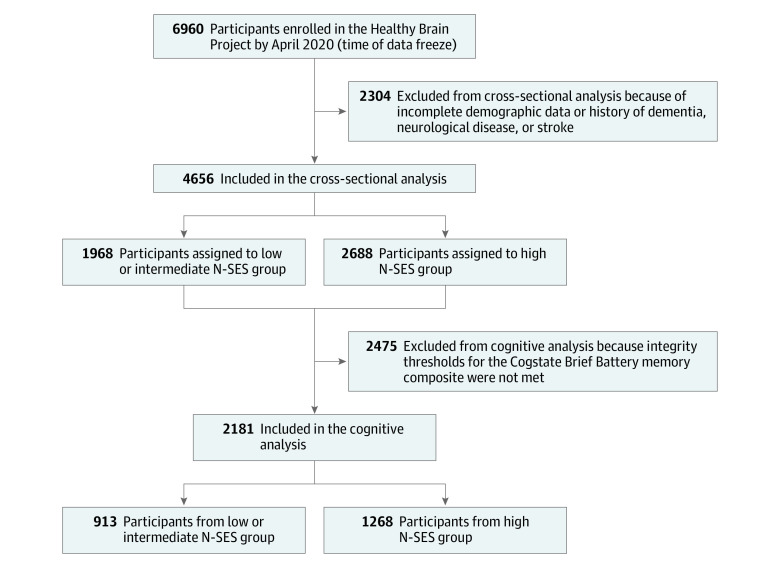
Participant Selection Neighborhood-level socioeconomic status (N-SES) was measured using the Index of Relative Socio-economic Advantage and Disadvantage, with deciles 1 to 7 indicating low to intermediate N-SES and deciles 8 to 10 indicating high N-SES.

Analyses were conducted using R software, version 1.2.5042 (R Foundation for Statistical Computing). Participants were excluded if they did not disclose their postcode of residence, age, sex, or years of education (Figure 1). All analyses were adjusted for age, sex, race (White vs racial minority group [African, Asian, Indigenous Australian, Latin American, Pacific Islander, or not specified]), years of education, and rurality (urban vs rural or regional). The only exception was that models including the original and modified CAIDE dementia risk scores as the outcome were not adjusted for variables that were already contained within the risk score (eg, models including the original CAIDE dementia risk score were not adjusted for age, sex, or years of education). Adjustment for race included only White and racial minority group categories because performing categorical analysis for each of the 7 categories of race would have been inappropriate when some categories were as small as 0.1% of the sample. Thus, it was more reasonable and statistically meaningful to categorize race as White (78.8% of the sample) and racial minority group (21.2%) for this cohort.

A series of individual linear regression analyses were conducted to examine the association of neighborhood-level SES (per decile unit increase) with the measures of cognition and the original and modified CAIDE dementia risk scores. A series of analyses of covariance were also conducted to assess whether performance on the cognitive tests differed between neighborhood-level SES categories. For these analyses, neighborhood-level SES was split at the median of 8.0; participants in deciles 8 to 10 were classified as advantaged and were compared with the remainder of the sample. The extent of difference between estimated means for each level of advantage was expressed as Cohen *d*. Interaction terms were added to the analysis of covariance models to examine whether any differences in cognition between neighborhood-level SES categories were modified by participant age, years of education (continuous variable), or CAIDE dementia risk score.

Sensitivity analyses were conducted to explore whether any associations between neighborhood-level SES and cognitive or dementia risk score outcomes were independent of personal SES. Scores from the Australian Socioeconomic Index 2006 were added as an additional covariate to the linear regression models examining the association between neighborhood-level SES and the outcomes. After applying the Benjamini and Hochberg^36^ method to control for false discovery rate, results were considered statistically significant at 2-tailed *P* < .05, with the exception of tests for interaction, which were considered significant at 2-tailed *P* < .10 after false discovery rate correction.

## Results

### Demographic Characteristics

**Figure 2.**
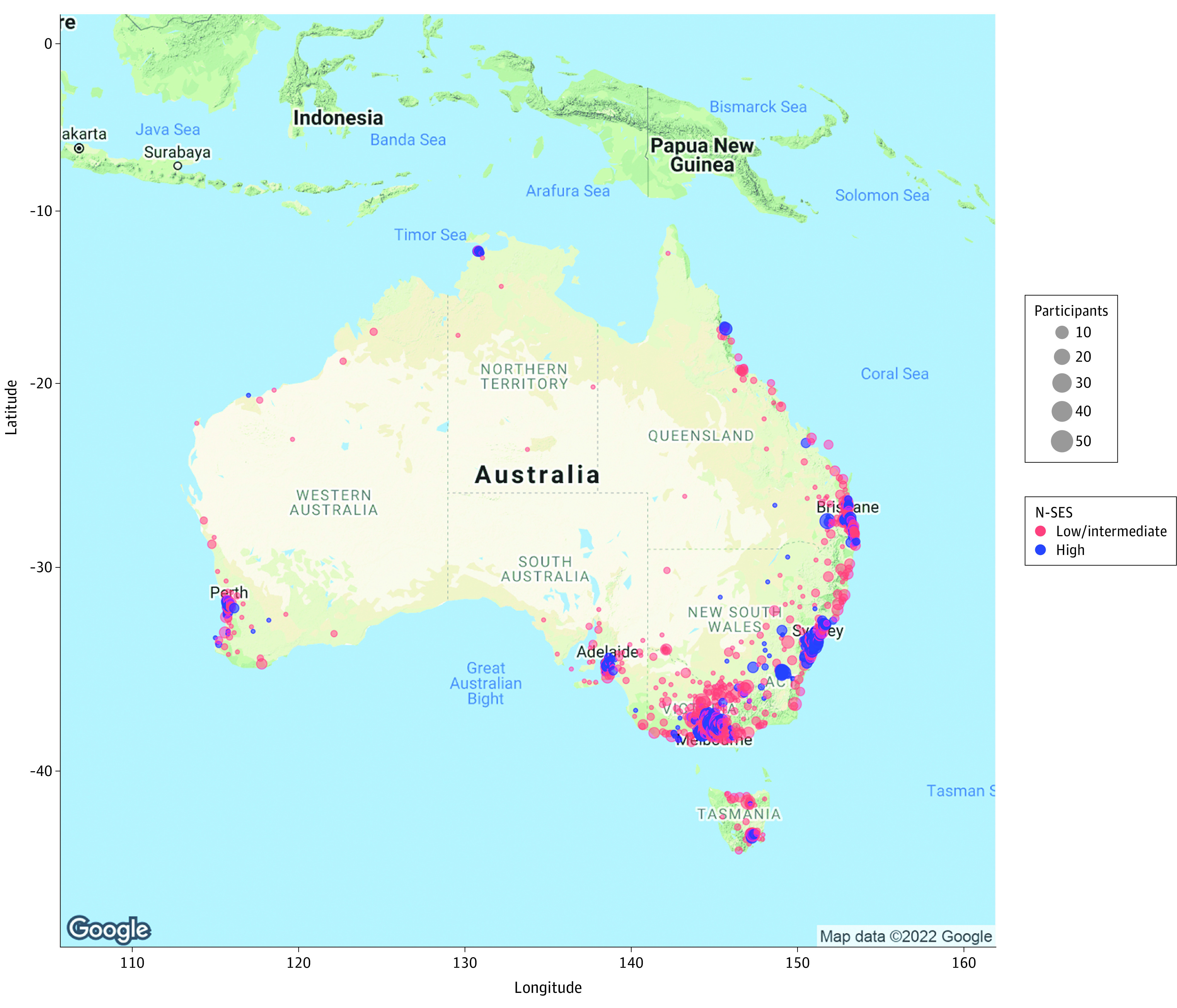
Geographic Distribution of Study Participants Across Australia by Neighborhood-Level Socioeconomic Status (N-SES) Neighborhood-level socioeconomic status was measured using the Index of Relative Socio-economic Advantage and Disadvantage, with deciles 1 to 7 indicating low to intermediate N-SES and deciles 8 to 10 indicating high N-SES. Google Maps API services were used to generate the map image and attributed coordinates to participant postcodes using R software, version 1.2.5042 (R Foundation for Statistical Computing). Google Maps content is subject to the Google Maps/Google Earth additional terms of service^38^ and the Google privacy policy^39^ as of February 1, 2022.

Among 4656 participants, the mean (SD) age was 56.1 (7.2) years; 3445 participants (74.0%) were female, and 1211 (26.0%) were male. A total of 6 participants (0.1%) identified as African, 121 (2.6%) as Asian, 57 (1.2%) as Indigenous Australian, 24 (0.5%) as Latin American, 9 (0.2%) as Pacific Islander, 3671 (78.8%) as White or European, and 768 (16.5%) indicated other race (not specified). Most participants were from the state of Victoria (2399 individuals [51.5%]); however, all other Australian states were represented, with 1263 participants (27.1%) residing in rural or regional Australia (Table 1). The geographic distribution of the sample is shown in Figure 2.

**Table 1.** Characteristics of the Study Population by Neighborhood-Level Socioeconomic Status

Characteristic	No./total No. (%)		
	Overall	Neighborhood-level SES	
		Low and intermediate^a^	High^b^
Total participants, No.	4656	1968	2688
Age, mean (SD), y	56.1 (7.2)	56.6 (7.2)	55.7 (7.2)
Sex			
Female	3445/4656 (74.0)	1480/1968 (75.2)	1964/2688 (73.1)
Male	1211/4656 (26.0)	488/1968 (24.8)	724/2688 (26.9)
Race			
African	6/4656 (0.1)	2/1968 (0.1)	4/2688 (0.1)
Asian	121/4656 (2.6)	40/1968 (2.0)	81/2688 (3.0)
Indigenous Australian	57/4656 (1.2)	24/1968 (1.2)	33/2688 (1.2)
Latin American	24/4656 (0.5)	8/1968 (0.4)	16/2688 (0.6)
Pacific Islander	9/4656 (0.2)	2/1968 (0.1)	7/2688 (0.3)
White or European	3671/4656 (78.8)	1518/1968 (77.1)	2154/2688 (80.1)
Other^c^	768/4656 (16.5)	375/1968 (19.1)	393/2688 (14.6)
Years of education, mean (SD), y	15.9 (3.5)	15.3 (3.5)	16.4 (3.4)
Income level^d^			
Higher	2772/4506 (61.5)	1064/1914 (55.6)	1708/2592 (65.9)
Lower	1734/4506 (38.5)	850/1914 (44.4)	884/2592 (34.1)
Current smoker	108/3821 (2.8)	58/1628 (3.6)	50/2193 (2.3)
BMI			
Median (IQR)	26.0 (6.4)	26.6 (7.3)	25.4 (6.0)
Obese (≥30)	863/3821 (22.6)	451/1628 (27.7)	412/2193 (18.8)
Diabetes	127/3821 (3.3)	60/1628 (3.7)	67/2193 (3.1)
Hypertension	748/3821 (19.6)	361/1628 (22.2)	388/2193 (17.7)
High cholesterol	679/3821 (17.8)	291/1628 (17.9)	388/2193 (17.7)
Rurality			
Urban	3393/4656 (72.9)	1000/1968 (50.8)	2393/2688 (89.0)
Rural or regional	1263/4656 (27.1)	968/1968 (49.2)	295/2688 (11.0)
PSS score >27^e^	38/4143 (0.9)	16/1753 (0.9)	22/2390 (0.9)
RAND SF-36 health-related QoL score, mean (SD)^f^	72.7 (19.6)	70.9 (20.2)	74.1 (19.1)
HADS score, mean (SD)^g^			
Depression	3.3 (2.9)	3.4 (3.0)	3.2 (2.9)
Anxiety	3.9 (3.4)	4.0 (3.5)	3.9 (3.3)
CAIDE score, mean (SD)^h^			
Original^i^	5.0 (2.3)	5.3 (2.3)	4.8 (2.3)
Modified^j^	1.4 (1.7)	1.6 (1.8)	1.2 (1.6)
AUSEI06 score, mean (SD)	66.3 (19.4)	63.4 (20.7)	68.4 (18.2)

Abbreviations: AUSEI06, Australian Socioeconomic Index 2006; BMI, body mass index (calculated as weight in kilograms divided by height in meters squared); CAIDE, Cardiovascular Risk Factors, Aging, and Incidence of Dementia; HADS, Hospital Anxiety and Depression Scale; PSS, Perceived Stress Scale; QoL, quality of life; RAND SF-36, RAND 36-Item Short Form Survey; SES, socioeconomic status.

^a^ Low and intermediate neighborhood-level SES defined as Index of Relative Socio-economic Advantage and Disadvantage deciles 1 to 7.

^b^ High neighborhood-level SES defined as Index of Relative Socio-economic Advantage and Disadvantage deciles 8 to 10.

^c^ Other races were not specified.

^d^ Higher income was defined as equal to or higher than the Australian median income of $51 389. Lower income was defined as lower than the Australian median income of $51 389.^37^

^e^ Score range, 0 to 40 points, with higher scores indicating higher perceived stress levels.

^f^ Score range, 0 to 100 points, with higher scores indicating more favorable state of health. Samples for the RAND SF-36 comprised 1240 participants overall, 524 participants with low or intermediate income, and 716 participants with high income.

^g^ Score range, 0 to 21 points for each subscale (anxiety or depression), with subscale scores greater than 8 points indicating anxiety or depression. Samples for the HADS comprised 4225 participants overall, 1792 participants with low or intermediate income, and 2433 participants with high income.

^h^ Samples for the CAIDE comprised 3235 participants overall, 1371 participants with low or intermediate income, and 1864 participants with high income.

^i^ Score range, 0 to 15 points, with higher scores indicating a higher risk of dementia.

^j^ Score range, 0-7 points, with higher scores indicating a higher risk of dementia. Calculated using physical activity, history of hypercholesterolemia, history of hypertension, and BMI only.

Across the sample, most participants (2688 [57.7%]) lived in areas with high neighborhood-level SES (IRSAD decile ≥8) vs low or intermediate neighborhood-level SES (1968 [42.3%]), with more individuals residing in urban areas (3393 [72.9%]) than rural or regional areas (1263 [27.1%]). Compared with participants living in areas with lower neighborhood-level SES, those in areas with high neighborhood-level SES were less than 1 year younger (mean [SD], 55.7 [7.2] years vs 56.6 [7.2] years), had slightly more formal education (mean [SD], 16.4 [3.4] years vs 15.3 [3.5] years), and consisted of 3.0 percentage points more White individuals (2154 [80.1%] vs 1518 [77.1%]) and 38.2 percentage points more individuals residing in urban areas (2393 [89.0%] vs 1000 [50.8%]) (Table 1). Participants living in areas with higher vs lower neighborhood-level SES also had a lower burden of most vascular risk factors (eg, hypertension: 388 of 2193 individuals [17.7%] vs 361 of 1628 individuals [22.2%]), higher health-related quality of life as measured by the RAND 36-Item Short Form Survey (mean [SD] score, 74.1 [19.1] points vs 70.9 [20.2] points), and fewer depressive symptoms as measured by the Hospital Anxiety and Depression Scale (mean [SD] score, 3.2 [2.9] points vs 3.4 [3.0] points). The proportion of men and women, levels of perceived stress, and anxiety symptoms were similar across neighborhood-level SES categories.

### Neighborhood-Level SES and Cognition

After adjustment for age, sex, race, years of education, and rurality, each decile unit increase in neighborhood-level SES was associated with better performance on the Cogstate Brief Battery memory composite (β [SE] = 0.022 [0.006]; *P* = .006) but not the attention composite (β [SE] = 0.009 [0.007]; *P* = .34). After the same adjustments, participants living in areas with neighborhood-level SES higher than the median (IRSAD decile ≥8) performed better on the memory composite (*F*_1-2172_ = 8.84; *P* = .01) compared with participants living in areas with neighborhood-level SES scores lower than the median (IRSAD deciles 1-7). The extent of difference between groups was small (Cohen *d* = 0.11 [95% CI, 0.03-0.26]) (Table 2). Higher and lower neighborhood-level SES groups did not differ in performance on the attention composite (*F*_1-2172_ = 0.70; *P* = .54).

**Table 2.** Association of Neighborhood-Level Socioeconomic Status With Cognition and Dementia Risk^a^

Variable	Attention^b^					Memory^c^					CAIDE dementia risk score^d^					Modified CAIDE dementia risk score^e^				
	*F* ^f^	*P* value	LSM (SE)	No.	Cohen *d* (95% CI)	*F* ^f^	*P* value	LSM (SE)	No.	Cohen *d* (95% CI)	*F* ^g^	*P* value	LSM (SE)	No.	Cohen *d* (95% CI)	*F* ^h^	*P* value	LSM (SE)	No.	Cohen *d* (95% CI)
Neighborhood-level SES group^i^	0.70	.54	NA	NA	NA	8.84	.01	NA	NA	NA	7.79	.01	NA	NA	NA	8.39	.01	NA	NA	NA
Age	207.18	<.001				9.44	.002				NA	NA				38.24	<.001			
Sex	0.80	.37				1.96	.16				NA	NA				12.83	<.001			
Race	0.56	.45				4.76	.03				5.74	.02				6.91	.009			
Years of education	5.33	.02				16.04	<.001				NA	NA				11.91	<.001			
Rurality	1.57	.21				1.64	.20				3.41	.07				0.19	.66			
Socioeconomic disadvantage	NA	NA	–0.01 (0.04)	912	0.03 (–0.07 to 0.15)	NA	NA	–0.12 (0.04)	912	0.11 (0.03 to 0.26)	NA	NA	0.14 (0.04)	912	0.11 (0.03 to 0.24)	NA	NA	0.16 (0.04)	912	0.10 (0.02 to 0.19)
Socioeconomic advantage			0.03 (0.04)	1268				0.02 (0.04)	1268				0.01 (0.04)	1268				0.03 (0.04)	1268	

Abbreviations: CAIDE, Cardiovascular Risk Factors, Aging, and Incidence of Dementia; LSM, least squares mean; SES, socioeconomic status.

^a^ Univariate multiple regression analysis included 2181 participants.

^b^ Attention was measured by composite *z* scores from the Cogstate Brief Battery detection and identification tasks.

^c^ Memory was measured by composite *z* scores from the Cogstate Brief Battery one card learning and one-back tasks.

^d^ CAIDE outcomes were not adjusted for age, sex, or years of education because these variables contribute to the CAIDE score itself.

^e^ Modified CAIDE dementia risk score was calculated using only physical activity, history of hypercholesteremia, history of hypertension, and body mass index.

^f^ df = 1 to 2172.

^g^ df = 1 to 2176.

^h^ df = 1 to 2173.

^i^ Socioeconomic status higher and lower than decile 8 on the Index of Relative Socio-economic Advantage and Disadvantage. *P* values were corrected for false discovery rate.

### Neighborhood-Level SES and Dementia Risk

Each decile unit increase in neighborhood-level SES was also associated with lower original CAIDE dementia risk scores (β [SE] = −0.070 [0.019]; *P* = .004) after adjustment for race and residential location. Results were comparable when including only modifiable risk factors in the CAIDE dementia risk score (β [SE] = −0.041 [0.014]; *P* = .01). Participants living in areas with neighborhood-level SES higher than the median (IRSAD decile ≥8) also had lower original (*F*_1-2176_ = 7.79; *P* = .01) and modified (*F*_1-2173_ = 8.39; *P* = .01) CAIDE dementia risk scores compared with participants in areas with neighborhood-level SES scores lower than the median (IRSAD deciles 1-7). The extent of difference between groups was small (original CAIDE: Cohen *d* = 0.11 [95% CI, 0.03-0.24]; modified CAIDE: Cohen *d* = 0.10 [95% CI, 0.02-0.19]) (Table 2).

### Age, Dementia Risk, and Education

**Figure 3.**
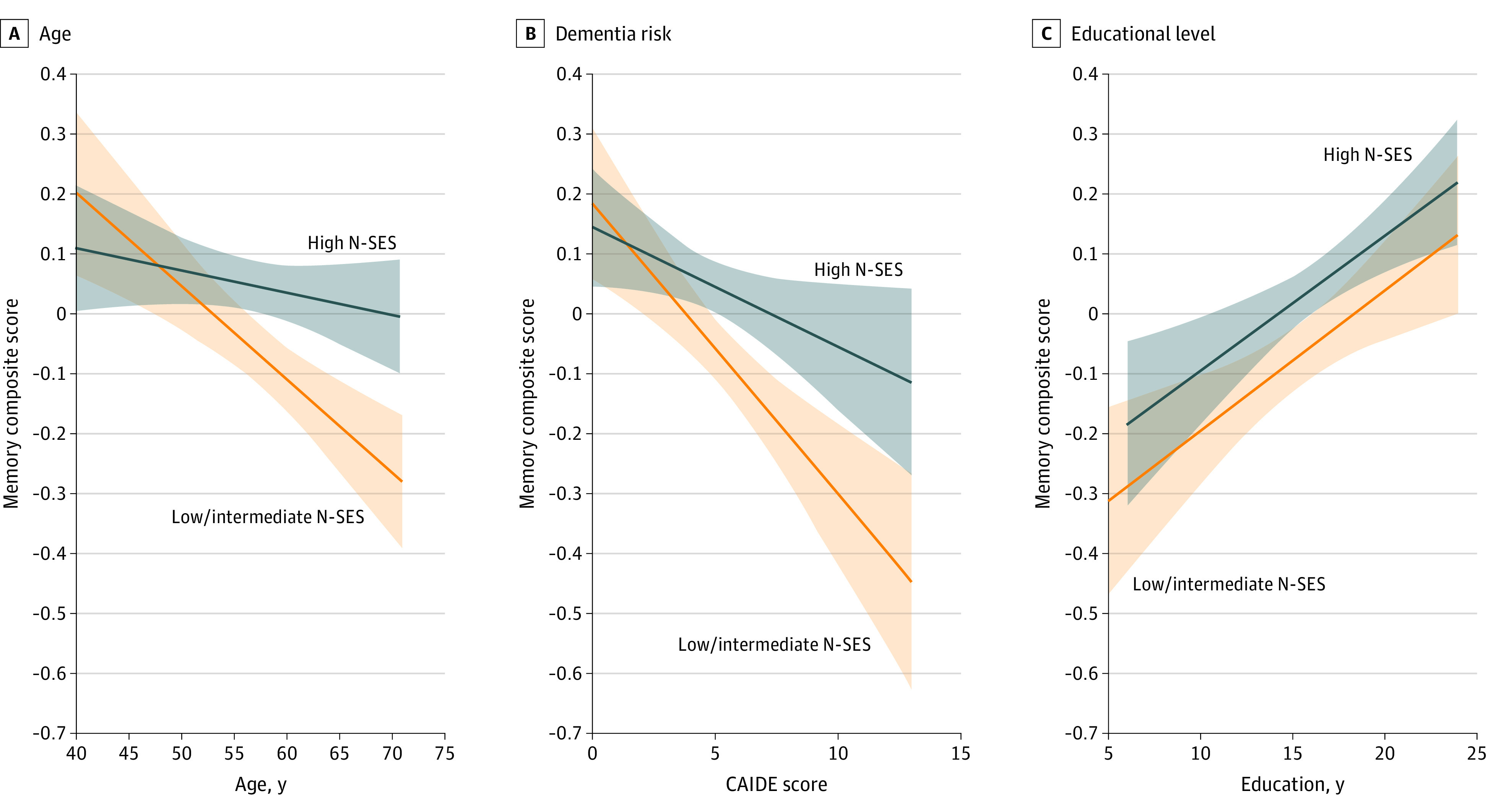
Association Between Memory Composite Score and Increasing Age, Dementia Risk Score, and Years of Education by Neighborhood-Level Socioeconomic Status (N-SES) A, Means were adjusted for sex, years of education, race and residential location. B, Original dementia risk score from the Cardiovascular Risk Factors, Aging, and Incidence of Dementia (CAIDE) tool. Means were adjusted for race and residential location. C, Means were adjusted for age, sex, race, and residential location. The Cogstate Brief Battery memory composite score was based on composite *z* scores from the one card learning and one back tests. Higher scores indicate a higher number of correct responses. Neighborhood-level socioeconomic status was measured using the Index of Relative Socio-economic Advantage and Disadvantage, with deciles 1 to 7 indicating low to intermediate N-SES (n = 913) and deciles 8 to 10 indicating high N-SES (n = 1268). Shaded areas indicate 95% CIs.

A significant interaction between neighborhood-level SES and age was observed for the memory composite (*F*_1-2171_ = 6.33; *P* = .02); differences in memory between neighborhood-level SES categories were larger among participants who were older (Figure 3A; eTable 3 in the Supplement). A significant interaction between neighborhood-level SES and the original CAIDE dementia risk score was also observed for the memory composite (*F*_1-2173_ = 4.02; *P* = .08), with differences in memory between neighborhood-level SES categories larger among participants who had higher original CAIDE dementia risk scores (Figure 3B; eTable 4 in the Supplement). No significant interaction was observed between neighborhood-level SES and years of education (*F*_1-2171_ = 0.01; *P* = .98) or between neighborhood-level SES and the modified CAIDE dementia risk score (*F*_1-2170_ = 1.14; *P* = .41) for the memory composite (Figure 3C; eTable 5 and eTable 6 in the Supplement). No effect modification was observed for the attention composite (eTables 3-6 in the Supplement).

### Sensitivity Analysis

Additional adjustment for personal SES did not substantially change the association between neighborhood-level SES and performance on the memory composite (β [SE] = 0.019 [0.007] per decile unit increase; *P* = .01) or the attention composite (β [SE] = 0.009 [0.008] per decile unit increase; *P* = .28). The association between neighborhood-level SES and the original CAIDE dementia risk score (β [SE] = −0.060 [0.020] per decile unit increase; *P* = .01) and the modified CAIDE dementia risk score (β [SE] = −0.041 [0.014] per decile unit increase; *P* = .01) also remained unchanged.

## Discussion

In this cross-sectional study, participants living in areas with higher neighborhood-level SES had better memory and lower dementia risk scores; however, neighborhood-level SES was not associated with attention. Differences in memory between individuals living in areas with higher vs lower neighborhood-level SES were larger among older adults and individuals with higher dementia risk scores. Together, these data suggest that persons in more disadvantaged neighborhoods generally have higher dementia risk scores and subtle differences in memory, even in midlife.

Numerous studies have reported an association between lower personal SES and a higher risk of dementia.^40,41^ However, few studies have investigated the association between neighborhood-level SES, cognition, and dementia risk, especially in midlife.^3^ Analyses of large cohorts in the US (12 599 participants) and the United Kingdom (6220 participants) found that low personal SES was associated with a higher risk of incident dementia, whereas neighborhood-level SES did not have an association.^41,42^ In contrast, low neighborhood-level SES has been associated with higher dementia incidence in Australia.^1^ In the US, an analysis of 447 samples across 2 Alzheimer disease research centers similarly found that living in the most disadvantaged neighborhood decile was associated with 2.18 higher odds of Alzheimer disease neuropathology.^43^ A cross-sectional analysis^44^ of data from the Wisconsin Registry for Alzheimer Prevention and Wisconsin Alzheimer Disease Research Center clinical cohort (951 participants aged 44-90 years) reported that participants living in the lowest 20% of the most disadvantaged neighborhoods had 4.1% lower hippocampal volume and 2.0% lower total brain volume compared with those living in all other neighborhoods. Cardiovascular risk mediated the association for total brain volume but not hippocampal volume.^44^ A longitudinal analysis (mean follow-up of 4.5 years) of the same cohort found that living in the lowest 20% of the most disadvantaged neighborhoods was also associated with cognitive decline and accelerated atrophy in regions of the brain affected by Alzheimer disease.^45^ Our study extends these data to a different context and setting. We found that neighborhood-level SES was also associated with better memory performance and lower dementia risk scores in an Australia-wide context across a large group of participants who were not confined to a single state or geographic subregion.

We observed that differences in memory between categories of neighborhood-level SES were greater at older ages and among participants with higher dementia risk scores. Notably, when limiting the CAIDE dementia risk score to only modifiable dementia risk factors, the risk score did not moderate the association between neighborhood-level SES and memory. These findings suggest that, in the present sample, the association between neighborhood-level SES and memory was moderated by nonmodifiable (eg, chronological age) rather than modifiable (eg, cardiovascular) dementia risk factors. Given that many cognitive functions decline with age, this finding might be explained by the relatively young age of the sample (40-70 years); that is, differences in memory between individuals living in different neighborhood-level SES areas may be more likely to emerge at older ages, when more brain atrophy and neuropathology are likely to be present. Although modifiable dementia risk factors did not moderate the association between lower neighborhood-level SES and worse memory, neighborhood-level SES deciles were independently associated with modified CAIDE dementia risk scores, a composite measure of physical inactivity, hypercholesterolemia, hypertension, and high body mass index. Thus, minimizing these dementia risk factors among individuals living in areas with lower neighborhood-level SES may help to lower dementia risk in old age without altering memory performance in midlife.

Beyond traditional dementia risk factors (eg, those factors included in the CAIDE dementia risk score), many potential mechanisms may have a role in the association of neighborhood-level SES with cognitive function (eg, access to health care; green space; walkability; air and noise pollution; number and quality of schools, libraries, and leisure centers; crime and perceptions of safety; and social disorder and cohesion). In addition, chronic stress over the life course and high levels of effort required to cope with multiple stressors may lead to a weathering (ie, early deterioration)^46,47^ of biological systems. Socioeconomically disadvantaged groups who experience structural disadvantage, discrimination, and social and economic adversity may experience greater physiological demand to compensate, leading to early morbidity and mortality.^46,47^ Furthermore, living in areas with low neighborhood-level SES has been associated with epigenetic changes, as observed through increased genetic methylation, including methylation of stress and inflammatory genes.^47,48^ Psychological stress and its biological correlate, cortisol, are associated with increased dementia risk because of the direct neurotoxic effects of cortisol and other potential inflammatory and metabolic neurodegenerative pathways.^49,50^ Therefore, there are a variety of proposed mechanisms through which neighborhood-level SES may impact dementia risk and cognition.

Comprehensive dementia prevention programs will require initiatives to address exposome dementia risk factors in disadvantaged communities to limit inequalities in dementia burden between advantaged and disadvantaged communities and to have a maximal impact for curtailing the increasing burden of dementia. Moreover, interventions may need to consider the potential influence of the neighborhood context in which an individual is situated. For example, an intervention designed to increase physical activity in someone living in a disadvantaged neighborhood may need to address potential barriers (eg, perceived safety) to increase physical activity levels.

### Strengths and Limitations

This study has strengths. The study involves an Australia-wide cohort with representation from rural and regional areas, which increases the generalizability of its results to the Australian population at large.

The study also has limitations. The HBP uses a convenience sample, meaning that there is an element of selection bias and overrepresentation of certain groups (eg, high neighborhood-level SES groups). Thus, it is unclear how our results will generalize to lower neighborhood-level SES contexts. For example, the extent of difference in cognition among participants living in areas higher and lower than the median (decile 8) of neighborhood-level SES scores may differ from the extent of difference among participants at the extreme ends of SES distribution in the wider population (eg, very disadvantaged vs very advantaged). Data on cardiovascular risk factors and covariates were self-reported and subject to some amount of reporting bias. Our study was cross-sectional, meaning that participants were assigned a neighborhood-level SES score based on the residential postcode provided at the time of data collection. This approach captured current neighborhood-level SES but may not have accurately estimated long-standing neighborhood-level SES among participants who changed residence throughout their lives. It would be interesting for future studies to consider current and former neighborhood-level SES classifications to investigate time lived in neighborhood-level SES areas and the moderating effect of dose or to explore sensitive periods across the lifespan. Moreover, the observational nature of our cross-sectional study precludes us from making inferences on causality.

## Conclusions

This cross-sectional study found that participants aged 40 to 70 years residing in lower neighborhood-level SES areas had worse memory and higher dementia risk scores. Future research is warranted to investigate whether strategies to improve health, particularly vascular health, in communities with lower SES can help to curtail dementia risk. Dementia prevention research and clinical trials may need to ensure that individuals living in lower SES areas are adequately represented, that interventions are accessible, and that the challenges related to living in those areas are addressed to increase the possibility of clinical impact on at-risk groups and the generalizability of findings to the broader community. In addition, the impact of social and public health policies designed to promote health equality will be an important avenue of future dementia prevention research.

## Notes

Supplement. **eMethods.** Abbreviations, Neighborhood-Level SES Estimations, Personal SES Estimations, and Measurement of Cognition **eTable 1.** List of Variables in the Index of Relative Socio-economic Advantage and Disadvantage **eTable 2.** CAIDE Dementia Risk Score Used in the Study and Number of Points **eTable 3.** Association Between Neighborhood-Level SES and Cognition With Interaction by Age **eTable 4.** Association Between Neighborhood-Level SES and Cognition With Interaction by CAIDE Dementia Risk Scores **eTable 5.** Association Between Neighborhood-Level SES and Cognition With Interaction by Years of Education **eTable 6.** Association Between Neighborhood-Level SES and Cognition With Interaction by Modified CAIDE Dementia Risk Scores
eReferences
