# Supplementary material for: Association of Neighborhood-Level Socioeconomic Measures With Cognition and Dementia Risk in Australian Adults
Source: JAMA Netw Open. 2022 Mar 25;5(3):e224071. doi: 10.1001/jamanetworkopen.2022.4071 (PMC8956972; doi:10.1001/jamanetworkopen.2022.4071)
Supplement: Supplement. — eMethods. Abbreviations, Neighborhood-Level SES Estimations, Personal SES Estimations, and Measurement of Cognition eTable 1. List of Variables in the Index of Relative Socio-economic Advantage and Disadvantage eTable 2. CAIDE Dementia Risk Score Used in the Study and Number of Points eTable 3. Association Between Neighborhood-Level SES and Cognition With Interaction by Age eTable 4. Association Between Neighborhood-Level SES and Cognition With Interaction by CAIDE Dementia Risk Scores eTable 5. Association Between Neighborhood-Level SES and Cognition With Interaction by Years of Education eTable 6. Association Between Neighborhood-Level SES and Cognition With Interaction by Modified CAIDE Dementia Risk Scores eReferences [file jamanetwopen-e224071-s001.pdf]

Supplementary Online Content

Pase MP, Rowsthorn E, Cavuoto MG, et al. Association of neighborhood-level socioeconomic measures with cognition and dementia risk in Australian adults. *JAMA Netw Open*. 2022;5(3):e224071. doi:10.1001/jamanetworkopen.2022.4071

eMethods. Abbreviations, Neighborhood-Level SES Estimations, Personal SES Estimations, and Measurement of Cognition

eTable 1. List of Variables in the Index of Relative Socio-economic Advantage and Disadvantage

eTable 2. CAIDE Dementia Risk Score Used in the Study and Number of Points

eTable 3. Association Between Neighborhood-Level SES and Cognition With Interaction by Age

eTable 4. Association Between Neighborhood-Level SES and Cognition With Interaction by CAIDE Dementia Risk Scores

eTable 5. Association Between Neighborhood-Level SES and Cognition With Interaction by Years of Education

eTable 6. Association Between Neighborhood-Level SES and Cognition With Interaction by Modified CAIDE Dementia Risk Scores

eReferences

This supplementary material has been provided by the authors to give readers additional information about their work.

© 2022 Pase MP et al. *JAMA Network Open*.

eMethods. Abbreviations, Neighborhood-Level SES Estimations, Personal SES Estimations, and Measurement of Cognition

Abbreviations.

AUSEI06: Australian Socioeconomic Index 2006

CAIDE: Cardiovascular Risk Factors, Aging and Dementia risk score

CBB: Cogstate Brief Battery

n-SES: neighbourhood socio-economic status

SES: socio-economic status

N-SES estimations.

The Australian Bureau of Statistics (ABS) developed the Index of Relative Socioeconomic Advantage and Disadvantage (IRSAD) using data from the 2016 Census. The IRSAD ranks neighborhoods throughout Australia from most disadvantaged to most advantaged based on the SES characteristics of the area.[1] The IRSAD is derived from a combination of 11 socioeconomic variables, such as income, education, unemployment rates, occupational skills, disability, vehicle ownership, internet connection, family structure (e.g., one parent with a dependent), and housing arrangements (see *eTable 1*). These variables are expressed by percentage (i.e. % of the population in an area that own a vehicle), and were weighted using Principal Component Analysis by the ABS to proportionately determine the index score per neighbourhood.[1] We used the residential postcode provided by each participant to derive an IRSAD score that ranked participants according to deciles of n-SES (higher deciles indicate greater advantage).

The IRSAD scores and deciles of n-SES are comparable to other measures that describe regions by socioeconomic variables used in other countries. For example, the Area Deprivation Index (ADI) was derived from American census data and similarly utilises the percentage of population and median data to score advantage from SES variables (including education, income, housing and household characteristics). Additionally, the European Deprivation Index (EDI) is used to describe a country-specific ecological deprivation index at a small area level, incorporating population statistics, income and living conditions.

Personal SES estimations.

Two authors independently coded participants' occupations into the Australian and New Zealand Standard Classification of Occupations (ANZSCO; ABS 2006), with any discrepancies resolved by consensus. Each occupation has a corresponding weighted AUSEI06 score.[3] Where possible, responses were coded into the level of the ANZSCO minor groups, otherwise, a superordinate category was used (i.e., sub-major or major groups). Each occupational group has a corresponding AUSEI06 value which was used as the measure of personal SES. For participants who had retired, we coded the AUSEI06 score using their last occupation prior to retirement.

© 2022 Pase MP et al. *JAMA Network Open*.

Measurement of cognition.

The CBB consists of four tests: Detection (DET), Identification (IDN), One Card Learning (OCL), and One-Back (OBK). Briefly, DET is a simple reaction time task shown to measure psychomotor function, and IDN is a choice reaction time task shown to measure visual attention. The primary outcome for DET and IDN was reaction time in milliseconds, with lower scores indicating faster task completion. OCL is a continuous visual recognition task set within a pattern separation model, and OBK is a task of working memory. The primary outcome for OCL and OBK was the proportion of correct responses, normalized using an arcsine square-root transformation, with higher scores indicating better performance. The Attention Composite was computed by standardizing and averaging the DET and IDN tests. As DET and IDN are speeded measures, they were reverse-scored such that negative values reflected poorer performance. The Memory Composite was computed by standardizing and averaging the OCL and OBK test scores. Tests were standardized using the baseline mean and standard deviation of the entire sample. Higher scores on both the Memory and Attention composites indicate better performance.

© 2022 Pase MP et al. *JAMA Network Open*.

eTable 1. List of Variables in the Index of Relative Socio-economic Advantage and Disadvantage[1]

Dimension	Variable	Summarised Definition
Income	Low Income	% people with stated annual household equivalised income between $1 and $25,999.
	High Income	% people with stated annual household equivalised income greater than $78,000
Education	No High School Competition	% people aged 15 years and over whose highest level of education is year 11 or lower
	No Education	% people aged 15 years and over who have no educational attainment
	Certificate	% people aged 15 years and over whose highest level of education is a certificate III or IV
	At University	% people aged 15 years and over at a university or tertiary institution
	Diploma	% people aged 15 years and over whose highest level of education is an advanced diploma or diploma
Employment	Unemployed	% people aged 15 years and over who are unemployed
Occupation	Labour	% employed people classified as labourers
	Driver	% employed people classified as machinery operators and drivers
	Service Worker	% employed people classified as low-skill community and personal service workers
	Sales Worker	% employed people classified as low-skill sales workers
	Manager	% employed people classified as managers
	Professionals	% employed people classified as professionals
Housing	Low Rent	% occupied private dwellings paying less than $215 per week in rent
	Overcrowded	% occupied private dwellings requiring one or more extra bedrooms
	High Rent	% occupied private dwellings paying more than $470 per week in rent
	High Bedrooms	% occupied private dwellings with four or more bedrooms
	High Mortgage	% occupied private dwellings paying more than $2,800 per month in mortgage repayments
Other	Unemployed w/ Children	% families with children under 15 years of age and jobless parents
	One Parent	% families that are one parent families with dependent offspring only
	Under 70 w/Disability	% people aged under 70 who need assistance with core activities
	No Car	% occupied private dwellings with no cars
	Separated/Divorced	% people aged 15 and over who are separated or divorced
	No Internet	% occupied private dwellings with no internet connection

Further detail of the calculation of variables can be found in the published SEIFA 2016 Technical Paper[1].

© 2022 Pase MP et al. *JAMA Network Open*.

eTable 2. CAIDE Dementia Risk Score Used in the Study and Number of Points[2]

Criteria	Original CAIDE Score (Points awarded)	Modified CAIDE Score (Points awarded)
Age, years		
<47	0	-
47-53	3	
>53	4	
Education, years		
≥10	0	-
7-9	2	
0-6	3	
Sex		
Women	0	-
Men	1	
Hypertension		
SBP ≤140 mmHg	0	0
SBP >140 mmHg	2	2
BMI, kg/m^2		
≤30	0	0
>30	2	2
Hypercholesterinemia		
≤6.5 mmol/L	0	0
>6.5 mmol/L	2	2
Physical activity		
Active	0	0
Inactive	1	1
	Max 15 points	Max 7 points

CAIDE: Cardiovascular Risk Factors, Aging and Dementia Study; SBP: systolic blood pressure; BMI: Body Mass Index. Components of Age, Education, Sex were scored using demographic data collected. Hypercholesterinemia was scored according to participant self-reports of high cholesterol diagnosis in the Healthy History questionnaires rather than quantitative blood work (without diagnosis substituted for ≤6.5 mmol/L and scored 0; with diagnosis substituted for >6.5 mmol/L and scored 2). Hypertension and BMI were also scored using data from Health History questionaries. Lifestyle relating to physical activity levels was captured using the International Physical Activity Questionnaire (IPAQ[4]). Physical activity was scored using the IPAQ 'Active' categorical score ("High" or "Moderate" activity levels corresponded to "Active" and scored 0; a "Low" activity level corresponded to "Inactive" and scored 1). The scores from each component were summed to acquire the CAIDE Dementia Risk total score.

© 2022 Pase MP et al. *JAMA Network Open*.

eTable 3. Association Between Neighborhood-Level SES and Cognition With Interaction by Age

	Attention		Memory	
	F(df)	*p*	F(df)	*p*
Advantage Group	0.695(1,2171)	.41	**8.539(1,2171)**	**.004**
Age	**84.169(1,2171)**	**<.001**	0.651(1,2171)	.42
Sex	0.797(1,2171)	.37	1.957(1,2171)	.16
Race	0.557(1,2171)	.46	**5.219(1,2171)**	**.02**
Education	**5.330(1,2171)**	**.02**	**15.638(1,2171)**	**<.001**
Rural/Urban	1.564(1,2171)	.21	1.650(1,2171)	.20
Advantage Group x Age[a]	0.001(1,2171)	.98	**6.332(1,2171)**	**.02**
	LSM (SE)	N	LSM (SE)	N
Disadvantage	-0.298 (0.032)	912	-0.131 (0.032)	912
Advantage	-0.299 (0.026)	1268	-0.022 (0.027)	1268
Cohen's d (95 % CI)	0.00 (-0.09, 0.08)		**0.11 (0.03, 0.20)**	

[a]FDR correct p value reported for outcome of interest
Univariate multiple regression. N = 2181.
Attention = composite Z scores of the Detection and Identification tests; Memory = composite Z scores of the One Card Learning and One Back tests; CAIDE = Cardiovascular Risk Factors, Aging and Incidence of Dementia – dementia risk score.

© 2022 Pase MP et al. *JAMA Network Open*.

eTable 4. Association Between Neighborhood-Level SES and Cognition With Interaction by CAIDE Dementia Risk Scores

	Attention		Memory	
	F(df)	*p*	F(df)	*p*
Advantage Group	0.992(1,2173)	.32	**10.097(1,2173)**	**.002**
Race	0.267(1,2173)	.61	**6.209(1,2173)**	**.01**
CAIDE	**47.835(1,2173)**	**<.001**	**3.966(1,2173)**	**.05**
Rural/Urban	0.333(1,2173)	.56	1.222(1,2173)	.27
Advantage Group x CAIDE[a]	0.487(1,2173)	.58	**4.022(1,2173)**	**.08**
	LSM (SE)	N	LSM (SE)	N
Disadvantage	-0.221 (0.032)	912	-0.142 (0.032)	912
Advantage	-0.191 (0.027)	1268	-0.056 (0.028)	1268
Cohen's d (95 % CI)	0.03 (-0.05, 0.12)		**0.09 (0.00, 0.17)**	

[a]FDR correct p value reported for outcome of interest
Univariate multiple regression. N = 2181.
Attention = composite Z scores of the Detection and Identification tests; Memory = composite Z scores of the One Card Learning and One Back tests; CAIDE = Cardiovascular Risk Factors, Aging and Incidence of Dementia – dementia risk score.

© 2022 Pase MP et al. *JAMA Network Open*.

eTable 5. Association Between Neighborhood-Level SES and Cognition With Interaction by Years of Education

	Attention		Memory	
	F(df)	*p*	F(df)	*p*
Advantage Group	0.678(1,2171)	.41	**8.816(1,2171)**	**.003**
Age	**207.103(1,2171)**	**<.001**	**9.432(1,2171)**	**.002**
Sex	0.815(1,2171)	.37	1.946(1,2171)	.16
Race	0.566(1,2171)	.45	**4.756(1,2171)**	**.03**
Education	3.327(1,2171)	.07	**7.744(1,2171)**	**.005**
Rural/Urban	1.572(1,2171)	.21	1.637(1,2171)	.20
Advantage Group x Education[a]	0.120(1,2171)	.81	0.007(1,2171)	.98
	LSM (SE)	N	LSM (SE)	N
Disadvantage	0.056 (0.031)	912	0.089 (0.032)	912
Advantage	0.042 (0.028)	1268	0.086 (0.029)	1268
Cohen's d (95 % CI)	0.01 (-0.07, 0.10)		**0.00 (-0.08, 0.09)**	

[a]FDR correct p value reported for outcome of interest
Univariate multiple regression. N = 2181.
Attention = composite Z scores of the Detection and Identification tests; Memory = composite Z scores of the One Card Learning and One Back tests; CAIDE = Cardiovascular Risk Factors, Aging and Incidence of Dementia – dementia risk score.

© 2022 Pase MP et al. *JAMA Network Open*.

eTable 6. Association Between Neighborhood-Level SES and Cognition With Interaction by Modified CAIDE Dementia Risk Scores

	Attention		Memory	
	F(df)	*p*	F(df)	*p*
Advantage Group	0.42(1,2170)	.52	**7.60(1,2170)**	**.005**
Age	**194.80(1,2170)**	**<.001**	**7.22(1,2170)**	**.007**
Sex	1.15(1,2170)	.28	1.41(1,2170)	.24
Mod CAIDE	**5.93(1, 2170)**	**.02**	**8.86(1, 2170)**	**.003**
Race	0.36(1,2170)	.55	**4.02(1,2170)**	**.045**
Education	**4.48(1,2170)**	**.03**	**14.31(1,2170)**	**<.001**
Rural/Urban	1.53(1,2170)	.22	1.59(1,2170)	.21
Advantage Group x Mod CAIDE[a]	0.64(1,2170)	.54	1.14(1,2170)	.41
	LSM (SE)	N	LSM (SE)	N
Disadvantage	0.001 (0.038)	912	-0.109 (0.040)	912
Advantage	0.031 (0.040)	1268	0.024 (0.041)	1268
Cohen's d (95 % CI)	0.02 (-0.06, 0.11)		**0.10 (0.01, 0.018)**	

[a]FDR correct p value reported for outcome of interest
Univariate multiple regression. N = 2181.
Attention = composite Z scores of the Detection and Identification tests; Memory = composite Z scores of the One Card Learning and One Back tests; Mod CAIDE = Modified Cardiovascular Risk Factors, Aging and Incidence of Dementia – dementia risk score computed only using physical activity, hypercholesterolemia, hypertension and BMI.

© 2022 Pase MP et al. *JAMA Network Open*.

eReferences

1. Australian Bureu of Statistics. Socio-Economic Indexes for Areas (SEIFA). Canberra: Australian Bureau of Statistics 2011.
2. Kivipelto M, Ngandu T, Laatikainen T, Winblad B, Soininen H, Tuomilehto J. Risk score for the prediction of dementia risk in 20 years among middle aged people: a longitudinal, population-based study. The Lancet Neurology 2006;5:735-741.
3. McMillan J, Beavis A, Jones FL. The AUSEI06:A new socioeconomic index for Australia. Journal of Sociology 2009;45:123-149.
4. Craig CL, Marshall AL, Sjostrom M, et al. International physical activity questionnaire: 12-country reliability and validity. Med Sci Sports Exerc 2003;35:1381-1395.

© 2022 Pase MP et al. *JAMA Network Open*.
